# Interfacial Modification of Mesoporous TiO_2_ Films with PbI_2_-Ethanolamine-Dimethyl Sulfoxide Solution for CsPbIBr_2_ Perovskite Solar Cells

**DOI:** 10.3390/nano10050962

**Published:** 2020-05-18

**Authors:** Xianwei Meng, Kailin Chi, Qian Li, Yu Cao, Gengxin Song, Bao Liu, Haibin Yang, Wuyou Fu

**Affiliations:** 1 State Key Laboratory of Superhard Materials, Jilin University, Changchun 130012, China; xwmeng17@mails.jlu.edu.cn (X.M.); yanghb@jlu.edu.cn (H.Y.); 2 School of Science, Northeast Electric Power University, Jilin 132012, China; 20182787@neepu.edu.cn (K.C.); songgengxin@neepu.edu.cn (G.S.); liubao@neepu.edu.cn (B.L.); 3 Beijing Key Lab of Cryo-Biomedical Engineering and Key Lab of Cryogenics, Technical Institute of Physics and Chemistry, Chinese Academy of Sciences, Beijing 100190, China; liqian@mail.ipc.ac.cn; 4 School of Electrical Engineering, Northeast Electric Power University, Jilin 132012, China; ycao@neepu.edu.cn

**Keywords:** all-inorganic perovskite, CsPbIBr_2_, solar cell, mesoporous structure, interfacial modification

## Abstract

As one of the most frequently-used electron-transporting materials, the mesoporous titanium dioxide (m-TiO_2_) film used in mesoporous structured perovskite solar cells (PSCs) can be employed for the scaffold of the perovskite film and as a pathway for electron transport, and the contact area between the perovskite and m-TiO_2_ directly determines the comprehensive performance of the PSCs. Because of the substandard interface combining quality between the all-inorganic perovskite CsPbIBr_2_ and m-TiO_2_, the development of the mesoporous structured CsPbIBr_2_ PSCs synthesized by the one-step method is severely limited. Here, we used a solution containing PbI_2_, monoethanolamine (EA) and dimethyl sulfoxide (DMSO) (PED) as the interfacial modifier to enhance the contact area and modify the m-TiO_2_/CsPbIBr_2_ contact characteristics. Comparatively, the performance of the solar device based on the PED-modified m-TiO_2_ layer has improved considerably, and its power conversion efficiency is up to 6.39%.

## 1. Introduction

Despite the advantages of simple fabrication processes, high conversion efficiency and successful techniques, the organic-inorganic perovskite solar cells (PSCs) under high humidity, high temperature and light conditions show poor stability [1,2,3,4,5,6], and since this poor stability seems unlikely to improve in the short term, scholars began researching ways to enhance the stability and efficiency of PSCs by investigating the all-inorganic hybrid PSCs [7,8,9]. As Cs^+^ ions have replaced organic molecules in organic-inorganic halide perovskites, the all-inorganic CsPbX_3_ (*X* = I or Br) materials present much better ultra-violet, thermal and humid stabilities in air [10,11,12,13]. Although CsPbI_3_ [14,15,16,17] and CsPbI_2_Br [18,19,20] materials exhibit acceptable light absorption capacity and performance with a relatively narrow band gap of 1.73 eV and 1.92 eV, respectively, both are difficult to be applied in practice due to their unstable structures, which easily lead to structure or performance degradation accompanied by color changes from dark red to yellow under laboratory conditions [10,13,15,21,22]. Many important physical properties of CsPbBr_3_ are known, such as high tolerance to humidity, light illumination [23] and a wide band gap of 2.30 eV [24,25]. However, extensive application in the field of photovoltaic is limited because of the low solubility of perovskite precursors (CsBr and PbBr_2_) in most organic solvents [26]. In contrast, CsPbIBr_2_ displays the best balance features for the all-inorganic PSCs as an absorption material in terms of stability and optical properties [27,28,29,30,31,32].

In mesoporous structured perovskite solar cells, the mesoporous TiO_2_ is an important electron transport material that has unique physical and chemical properties; it not only supplies the pathway for electron transport but also contacts the perovskite material to enhance the separation rate of photoinduced charges. Moreover, the interface relationships of the m-TiO_2_ exert a significant influence on the performance of PSCs. Many researchers have been focused on interfacial engineering to improve the comprehensive performance of the m-TiO_2_ films and have achieved many results in theory and application in the PSCs field. Briefly, the use of the interfacial modifier can effectively boost the performance and photostability of PSCs. In addition, all-inorganic CsPbIBr_2_ PSCs could be fabricated either on planar or mesoporous structures, but the former have received more research concentration on stable power output and the latter less. To our knowledge, TiO_2_ is the only mesoporous material used, and CsPbIBr_2_ films can only be synthesized by the only two-step solution method to date [26,33,34].

Herein, we successfully fabricated the CsPbIBr_2_ PSCs on a mesoporous structure, and the perovskite films were synthesized by a typical one-step method. The PED solution was used as the interfacial modifier to modify the m-TiO_2_/CsPbIBr_2_ interface and promote the precursor solution of CsPbIBr_2_ further diffusing into the grain boundaries of m-TiO_2_ film. Therefore, using PED on the interface can not only restrain the interface recombination to enhance the electronic transmission capability but can also promote CsPbIBr_2_ to fill the space in m-TiO_2_ film to enhance the interface combination. Compared with the standard mesoporous CsPbIBr_2_ PSCs we synthesized, the devices with PED modification presented a better performance with a power conversion efficiency (PCE) of 6.39%.

## 2. Experiment

### 2.1. Device Fabrication

Fluorine-doped tin oxide glass substrates (FTO, 6Ω/□) were patterned with a laser etcher (OPV Tech New Energy Co., Ltd., Yingkou, China.) and cleaned sequentially by a neutral detergent, deionized water, acetone, isopropanol and ethanol by ultrasound treatment. After being dried in the air, the FTO substrate was further cleaned in an ultraviolet treatment for 15 min, and then the TiO_2_ compact layer (c-TiO_2_) was spin-coated on the pre-conditioned FTO substrate according to the previous report [35]. Afterwards, the mesoporous TiO_2_ layer (m-TiO_2_) (Dysol, 30NR, diluted with ethanol at a ratio of 1:8, *w*/*w*) was coated on the FTO/c-TiO_2_ substrate by spin-coating at 5000 rpm for 30 s. After the film was dried on a hotplate at 125 °C for 5 min followed by the muffle furnace at 500 °C for 30 min, the pre-coated substrates were attained.

The following synthetic processes were carried out in a glove box under the highly purified argon environment. PED solution, which contained 2 mL DMSO (99.8%, Aladdin, Shanghai, China), 0.5 mL EA (double distillation, Aladdin, Shanghai, China) and 0.25 M PbI_2_ (99.99%, Xi’an p-OLED, Xi’an, China) was heated on a hot plate at 150 °C for 30 min. While the PbI_2_ dissolved completely, the PED was spin-coated on the surface of the cooled pre-coated substrates at 5000 rpm for 60 s and then heated and naturally cooled once again. For CsPbIBr_2_, the precursor solution prepared by full dissolving 260 mg CsI and 370 mg PbBr_2_ in 1 mL DMSO at 150 °C was coated on the PED film by spin-coating at the 5000 rpm for 60 s and annealed at 280 °C for 10 min subsequently. After Spiro-OMeTAD (>99%, OPV Tech New Energy Co., Ltd., Yingkou, LN, China) was spin-coated onto the CsPbIBr_2_ films at 3000 rpm for 30 s, the samples were removed from the glove box, and a 100 nm thick Ag was t deposited by thermal evaporation on the top of the Spiro-OMeTAD layer as metal electrode to complete the solar energy devices.

### 2.2. Device Characterizations

The crystal structures and composition of the synthesized samples were identified by X-ray diffraction (XRD, Cu Kα radiation, *λ* = 1.5418 Å, Rigaku D/max2500). The morphologies and structures of the films and solar devices were observed by a scanning electron microscope (SEM, FEI MAGELLAN 400, FEI, Hillsboro, OR, USA), and an energy dispersive spectroscope (EDS) attached to the SEM column was used to analysis the element composition of the corresponding samples. X-ray photoelectron spectroscopy (XPS) spectra were measured using ESCALAB 250Xi (Thermo Scientific, Waltham, MA, UK). A UV-Vis spectrometer (UV-3600, Shimadzu, TKY, Japan) was utilized to measure the absorption spectrum at a range of 300 nm to 800 nm. The steady-state photoluminescence (PL) spectrum was collected on a Ramascope System 1000 (*λ*_ex_ = 633 nm). The current-voltage (J-V) characteristics and external quantum efficiency (EQE) of the fabricated solar devices were measured by a solar cells test system (XP3000, Sanyou, Beijing, China) and an external quantum efficiency (EQE) measured system (Solar Cell Scan100, Zolix, Beijing, China), respectively.

## 3. Results and Discussion

### 3.1. X-ray Diffraction Studies and Compositional Analysis

**Figure 1:**
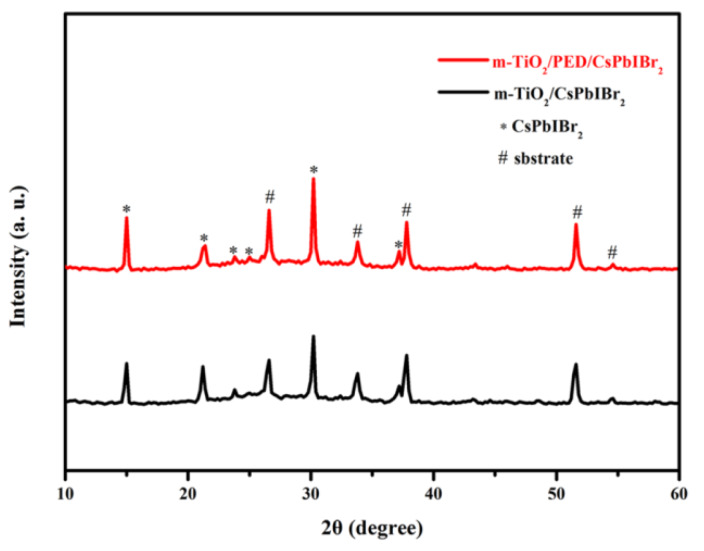
XRD patterns of m-TiO_2_/CsPbIBr_2_ films with and without PED modification.

Figure 1 shows the XRD patterns of CsPbIBr_2_ films synthesized on c-TiO_2_/m-TiO_2_ substrates with and without PED modification. Both patterns show the main diffraction peaks at 2*θ* of 15.00°, 21.40°, 30.19° and 37.20° corresponding to (111), (110), (200) and (211) planes of α-phase perovskite CsPbIBr_2_ [29,36,37], which confirms that both CsPbIBr_2_ films synthesized by the one-step method are pure, that the PED modification has no influence on the perovskite phase or crystallinity and that no PbI_2_ diffraction peak can be observed. However, as the PED solution is spin-coated on c-TiO_2_/m-TiO_2_ substrates without CsPbIBr_2_ film, a PbI_2_ characteristic peak at 12.60° can be found in the XRD pattern, and the I/Pb atomic ratio is approximate to 2:1, as confirmed from EDS spectra (Figure S1a,b respectively, Supporting Information), which means that PbI_2_ presents in the m-TiO_2_ film as crystals. Because of being easily soluble in DMSO, PbI_2_ in the PED-modified m-TiO_2_ film is redissolved in the perovskite precursor solution during the successively synthetic process of CsPbIBr_2_, which explains why no PbI_2_ diffraction peak is observed in Figure 1.

### 3.2. Morphological Characterization 

**Figure 2:**
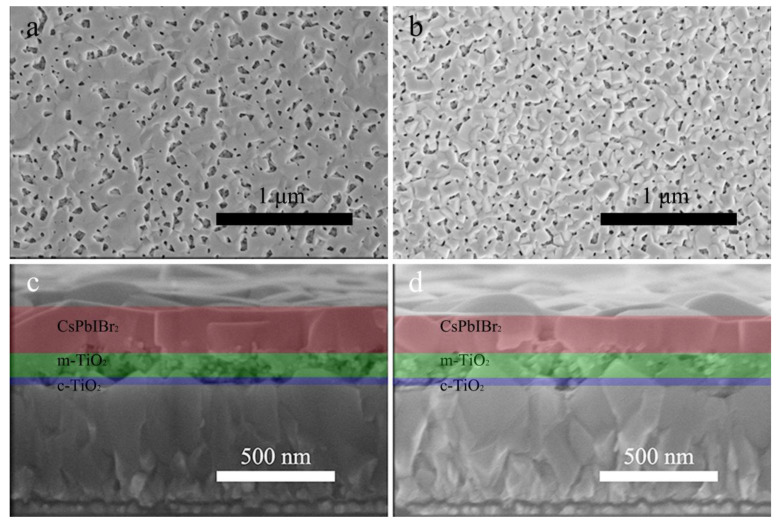
Top-view SEM images and cross-sectional SEM images of m-TiO_2_/CsPbIBr_2_ films (**a**,**c**) without and (**b**,**d**) with PED.

The influences of PED on morphologies of m-TiO_2_ and CsPbIBr_2_ were investigated by using SEM as shown in Figure 2. As demonstrated in Figure 2a, CsPbIBr_2_ film deposited on m-TiO_2_ shows a relatively porous attribute with non-uniform distribution but a smooth surface (Figure 2a). Apparently, compared with Figure 2a, the rough CsPbIBr_2_ film deposited on PED-modified m-TiO_2_ shown in Figure 2b is composed of almost homogeneous nanoparticles and pores that are uniformly distributed in the film. Because of the poor solubility of PbBr_2_ in the perovskite precursor solution, such abundant pores in the CsPbIBr_2_ film synthesized by the one-step method have seemed inevitable [26]. As illustrated in Figure 2c, pores can be clearly seen in the m-TiO_2_ film, and the thickness of the CsPbIBr_2_ film is about 210 nm. The cross-sectional SEM image of the PED-modified sample in Figure 2d shows that the perovskite film with a thickness of 160 nm is thinner than the pristine one and that the m-TiO_2_ film is almost filled up and only a very small number of pores remain. No interface between CsPbIBr_2_ and m-TiO_2_ layers can be clearly distinguished. Combined with the conclusions of XRD, we can confirm that the abundant perovskite precursor solution diffuses into the m-TiO_2_ film during the spin-coating process and generates CsPbIBr_2_ to fill up the pores, which is conductive to the transportation and collection process for the carriers [38,39].

### 3.3. XPS Analysis

**Figure 3:**
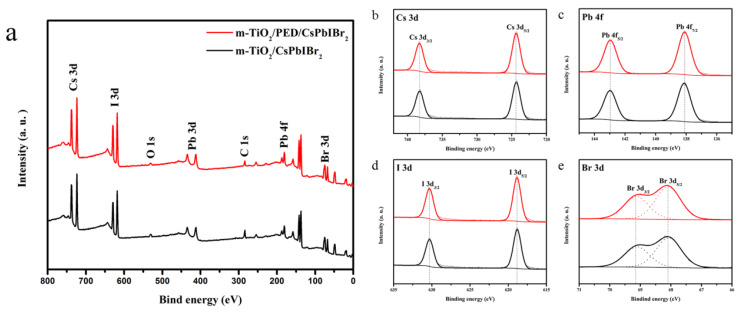
(**a**) XPS spectra and (**b**) Cs 3d, (**c**) Pb 4f, (**d**) I 3d and (**e**) Br 3d XPS core spectra of the m-TiO_2_/CsPbIBr_2_ films with and without PED modification.

XPS was utilized to probe the surficial elemental composition of CsPbIBr_2_ films with and without PED modification taking C 1s (284.8 eV) as the calibration. As seen from Figure 3a, obviously, the characteristic peaks of Cs, Pb, I, Br, C and O are detected, and no other element can be identified in each film. Figure 3b–e displays the XPS core spectra of corresponding Pb 4f, I 3d, Br 3d and Cs 3d, respectively. All of the binding energy peaks belonging to CsPbIBr_2_ constituent elements are in accordance with previous reports [36], and no notable peak shift can be observed for each of elements. The XPS results indicate that the existence or non-existence of PED has no essential influence on the stoichiometric of the CsPbIBr_2_ perovskite.

### 3.4. Optical Properties and Photovoltaic Performances

**Figure 4:**
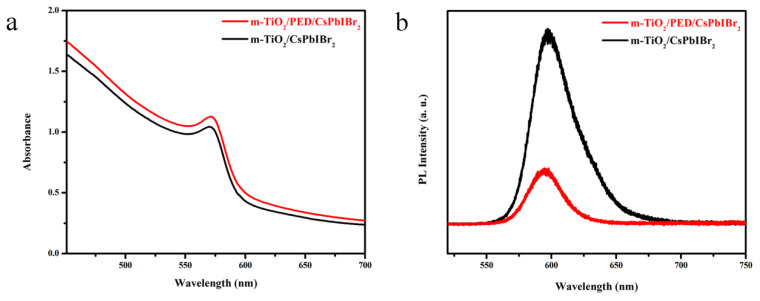
(**a**) UV-vis absorbance spectra and (**b**) photoluminescence (PL) spectra of the m-TiO_2_/CsPbIBr_2_ films with and without PED modification.

The photophysical properties of m-TiO_2_/CsPbIBr_2_ films with and without PED modification were then studied. Figure 4a presents their absorption spectra. Both the films exhibit similar absorption profiles in the visible region. The absorbance onset for the modified sample exhibits a slight red shift from about 606 to 610 nm, and the absorption intensity increases slightly in the whole measuring range compared with the pristine sample. Correspondingly, the calculated bandgap decreases from 2.09 to 2.08 eV (Figure S2, Supporting Information), which coincides with the relevant literature [40,41]. The PL spectra are demonstrated in Figure 4b, which signifies that when the peak position of the m-TiO_2_/CsPbIBr_2_ films with PED is blue-shifted (594.9 vs 597.6 nm), the luminous intensity also distinctly decreased. Such observations suggest that the trap states of the porous TiO_2_ layers that are full-filled by CsPbIBr_2_ and the spontaneous radiative recombination of CsPbIBr_2_ are passivated due to the interfacial modifier, PED, hence leading to the improvement of the charge separation and transfer process at the m-TiO_2_/CsPbIBr_2_ interface.

**Figure 5:**
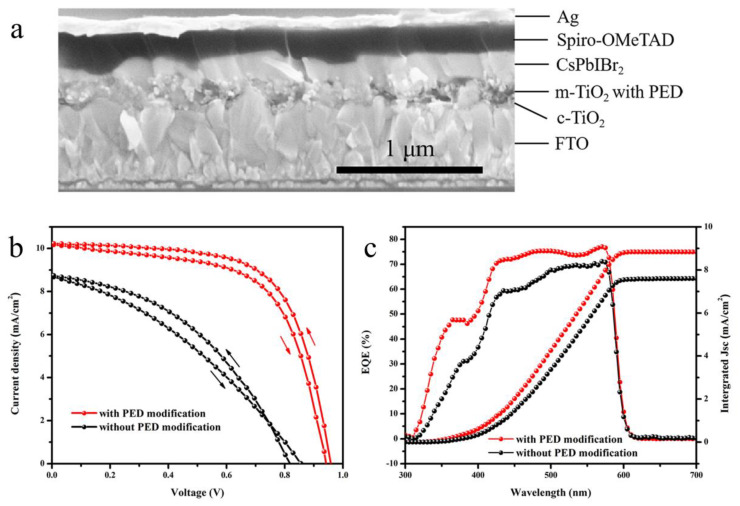
(**a**) Cross-sectional SEM image of the modified CsPbIBr_2_ devices. (**b**) *J-V* curves and (**c**) EQE spectra and the integrated product of the EQE curve of the corresponding devices.

The PSCs we successfully synthesized were based on the simple architecture of c-TiO_2_/m-TiO_2_/PED (or not)/CsPbIBr_2_/Spiro-OMeTAD/Ag, and the cross-section of the complete modified device is given in Figure 5a. Figure 5b provides the reverse- and forward-scanned *J-V* curves of two representative devices measured under simulated AM 1.5G illumination. The hysteresis index (*HI*) was applied to assess the hysteresis effect of the devices, which can be well defined according to the following equation [42]:

$$HI=\frac{PCEreverse–PCEforward}{PCEreverse}$$

Compared with the pristine device, the m-TiO_2_/PED device shows an expected slighter hysteresis behavior (a decrease from 13.8% to 7.2%), and all photovoltaic parameters including short circuit current density (*J_SC_*), open circuit voltage (*V_OC_*), fill factor (FF) and PCE are enhanced evidently. As listed in Table 1, the PED modification of m-TiO_2_ has significantly increased the *J_SC_* from 8.69–8.74 mA∙cm^−2^ to 10.22–10.28 mA∙cm^−2^, *V_OC_* from 0.82–0.86 V to 0.94–0.96 V and FF from 0.35–0.43 to 0.62–0.65, leading to an efficiency improvement of more than 110% from 2.62–3.04% to 5.93–6.39%. The EQE spectra and the corresponding integral current densities are provided in Figure 5c. Both EQE spectra present the same onsets at the wavelength of about 600 nm, which are roughly consistent with the results of absorption spectrum. A significant enhancement of EQE values is observed for the m-TiO_2_/PED device in the light absorption wavelength range of 300–575 nm, which is consistent with the increment in *J_SC_*. Meanwhile, the integrated current densities from EQE are close to the results of *J_SC_*, while the discrepancy mainly originates from the spectral mismatch between the solar simulator and EQE measurement system.

### 3.5. Possible Mechanism

**Figure 6:**
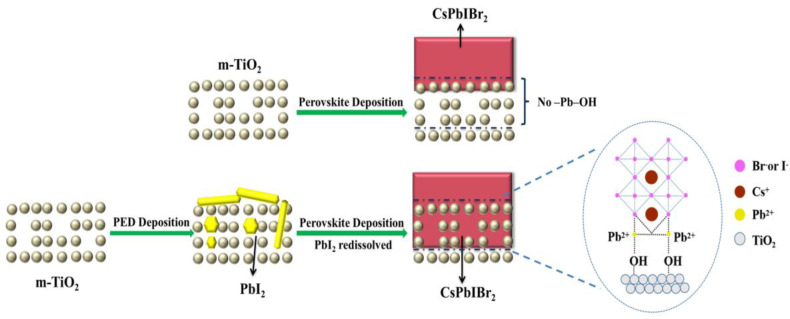
Schematic diagram illustrating the deposition of CsPbIBr_2_ perovskite on m-TiO_2_. The upper row shows the normal procedure to synthesize CsPbIBr_2_ film on m-TiO_2_. The lower row is the CsPbIBr_2_ deposition on the PED/m-TiO_2_ substrate.

The enhancement of power conversion efficiency of the CsPbIBr_2_ device was mainly ascribed to the PED solution, which contained proper EA and PbI_2_. The –OH group of EA within the PED solution would interact with PbI_2_ colloid (such as PbI_3_^−^ and PbI_4_^2−^) to form stable chemical bonding –OH–Pb [43]. As shown in Figure 6, while PED was spin-coated on m-TiO_2_, the unreacted PbI_2_ was forced to penetrate into the TiO_2_ mesoporous film by –OH group [44]. While the CsPbIBr_2_ precursor solution was coated on the m-TiO_2_/PED substrate, PbI_2_ in the mesoporous film provided the paths for the precursor solution diffusing into the pores while –OH–Pb coordinated to the TiO_2_ surface to improve the interface contact with CsPbIBr_2_ [43]. Therefore, the holes and chink were packed and closed by CsPbIBr_2_ in the annealing process, leading to the decreasing thickness of the CsPbIBr_2_ film on the m-TiO_2_/PED substrate. It should be noted that the surface color of m-TiO_2_/PED gradually converted from light yellow into colorless during the spin-coated process of the CsPbIBr_2_ precursor solution. This phenomenon was caused by the fact that PbI_2_ deposited in porous TiO_2_ was redissolved in the perovskite precursor solution and participated in the CsPbIBr_2_ growth and film-forming processes, leaving native defects due to nonstoichiometry [45,46]. That explains why the diffraction peak of PbI_2_ could not be found in the XRD pattern and why the morphology of the CsPbIBr_2_ surface also changed for the m-TiO_2_/PED/ CsPbIBr_2_ film. Furthermore, the improvement of the optical properties of the modified sample based on UV-Vis spectra and EQE spectra was mainly attributable to the CsPbIBr_2_ filling behavior and the stoichiometric change in the mesoporous film. The pores filled by perovskite in m-TiO_2_ could effectively increase the contact area between TiO_2_ nanoparticles and CsPbIBr_2_. The –OH–Pb group was advantageous for enhancing the interface contact, reducing trap states of the perovskite film, improving the electron extraction and charging transport rates [47,48], as well as restraining the recombination of charge carriers at the interface to help decrease the hysteresis of CsPbIBr_2_ device, as proved by the PL and J-V measurements.

## 4. Conclusions

In this work, we have successfully utilized PED solution as the interfacial modifier to modify the c-TiO_2_/m-TiO_2_ substrates, and the influence of PED on the CsPbIBr_2_ PSCs, which were synthesized by the one-step method on the mesoporous structure, was studied. By using a PED solution, CsPbIBr_2_ efficiently fills the pores of the m-TiO_2_ to enhance the m-TiO_2_/CsPbIB_2_ interfacial contact area and to optimize the contact characteristics due to the combination of PbI_2_ with EA. The PCE of the modified solar device has been significantly promoted from about 3.0% to over 6.3%, with an enhancement of 110% compared with the pristine one. The –OH–Pb group in PED is the main reason for the improvement of optical properties and the optimization of comprehensive performance for the modified device.

## Figures and Tables

**Table 1:** Photovoltaic parameters of the CsPbIBr_2_ devices with and without PED modification.

Substrate	Scan	*J_sc_* (mA∙cm^−2^)	*V_oc_* (V)	FF	PCE (%)
Without PED	forward	8.69	0.86	0.35	2.62
	reverse	8.74	0.82	0.43	3.04
With PED	forward	10.28	0.94	0.62	5.93
	reverse	10.22	0.96	0.65	6.39

## Supplementary Materials

The following are available online at https://www.mdpi.com/2079-4991/10/5/962/s1, Figure S1: (**a**) XRD pattern and (**b**) EDS spectra of the m-TiO_2_/PED film; Figure S2: (αhν)^2^ vs. hν plots of the m-TiO_2_/CsPbIBr_2_ with and without PED modification.
