# Supplementary material for: Interfacial Modification of Mesoporous TiO2 Films with PbI2-Ethanolamine-Dimethyl Sulfoxide Solution for CsPbIBr2 Perovskite Solar Cells
Source: Nanomaterials (Basel). 2020 May 18;10(5):962. doi: 10.3390/nano10050962 (PMC7325578; doi:10.3390/nano10050962)
Supplement: Supplementary file 1 [file nanomaterials-10-00962-s001.pdf]

Supplementary Material

Interfacial Modification of Mesoporous TiO_2 Films with PbI_2-Ethanolamine-Dimethyl Sulfoxide Solution for $CsPbIBr_2$ Perovskite Solar Cells

Xianwei Meng [1], Kailin Chi [2], Qian Li [3], Yu Cao [4], Gengxin Song [2], Bao Liu [2], Haibin Yang [1] and Wuyou Fu [1,*]

[1] State Key Laboratory of Superhard Materials, Jilin University, Changchun 130012, China; xwmeng17@mails.jlu.edu.cn (X.M.); yanghb@jlu.edu.cn (H.Y.)

[2] School of Science, Northeast Electric Power University, Jilin 132012, China; 20182787@neepu.edu.cn (K.C.); songgengxin@neepu.edu.cn (G.S.); liubao@neepu.edu.cn (B.L.)

[3] Beijing Key Lab of Cryo-Biomedical Engineering and Key Lab of Cryogenics, Technical Institute of Physics and Chemistry, Chinese Academy of Sciences, Beijing 100190, China; liqian@mail.ipc.ac.cn

[4] School of Electrical Engineering, Northeast Electric Power University, Jilin 132012, China; ycao@neepu.edu.cn

* Correspondence: fuwy@jlu.edu.cn; Tel.: +86-431-8516-8763-801; Fax: +86-431-8516-8763-801

Received: 27 April 2020; Accepted: 14 May 2020; Published: 18 May 2020

Results and discussion

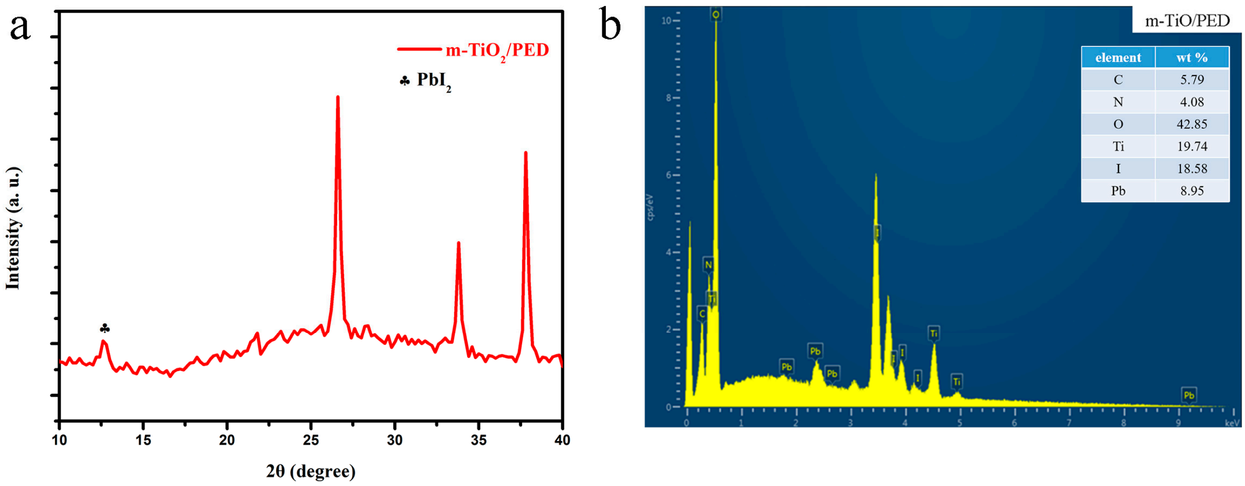

Figure S1. a) XRD pattern and **b**) EDS spectra of the m-TiO_2/PED film.

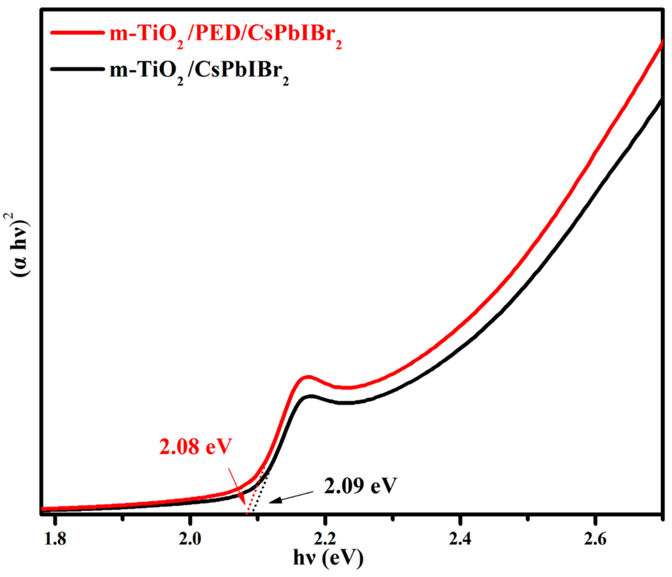

Figure S2. $(\alpha h\nu)^2$ vs hν plots of the m-TiO_2/$CsPbIBr_2$ with and without PED modification.
